# Esketamine for resistant depression in older people with cognitive impairment: a case report

**DOI:** 10.1192/j.eurpsy.2024.1103

**Published:** 2024-08-27

**Authors:** F. Mazzoni, V. Arienti, S. C. Civardi, G. Carnevale Miacca, P. Leali, F. Santilli, A. Guffanti, N. Brondino, M. Olivola

**Affiliations:** Department of Brain and Behavioral Sciences, University of Pavia, Pavia, Italy

## Abstract

**Introduction:**

Depression represents a significant challenge in terms of disability among the elderly population and its responsiveness to conventional treatment approaches tends to diminish in this population group. Esketamine has shown both effectiveness and safety in addressing treatment-resistant depression in older patients.

**Objectives:**

Currently, there is a lack of available literature regarding the use of esketamine in the treatment of patients experiencing both cognitive decline and treatment-resistant depression (TRD). We administered esketamine to a 79-year- old patient to evaluate the effectiveness and tolerance of the medication.

**Methods:**

We recruited a 79 year old female referred to the outpatient clinics of Pavia suffering from TRD with current Severe Depressive Episode (scoring 42 on the MADRS) with cognitive impairment (MMSE 16/30). The patient was on a fourth-line treatment. First-line treatment was started with paroxetine 40 mg from september 2021 to May 2022, switched to sertraline 50 mg. Second-line treatment with quetiapine 150 mg from June 2022 to December 2022 failed, despite optimal compliance for both lines of treatment. Then third line treatment with fluoxetine 20 mg, olanzapine 10 mg was initiated from December 2022 to May 2023. Study duration was 12 weeks. Anamnestic data and psychometric (MADRS, HAMD-21, HAM-A) and cognitive (MMSE and MoCA TEST) assessment were collected from medical records at baseline (T0), one month (T1) and three month (T2) follow-ups.

**Results:**

MADRS, HAM-A and HAM-D values decreased significantly at T1 and T2 follow-ups. T0: MADRS 42, HAM-D 33, HAM-A 54; T1: MADRS 18, HAM-D 12, HAM-A 15; T2: MADRS 4, HAM-D 5, HAM-A 10. We also observed an improvement in cognitive test: T0: MMSE 16/30, MoCA test 4/30; T1: MMSE 18/30, MoCA test 6/30; T2: MMSE 20/30, MoCA test 8/30. The patient reported one episode of hypertension treated with clonidine after two moth of treatment, and mild prolonged motor slowing lasting about two hours after esketamine in the first month.

**Conclusions:**

This case documented a successful treatment using intranasal esketamine in combination with an SSRI (Fluoxetine) for an older individual with cognitive impairment and a persistent anxiety-depressive syndrome. This approach was employed as a therapeutic intervention after multiple unsuccessful attempts with other antidepressant medications. Our findings confirmed the safety and tolerability of esketamine in an elderly female with cognitive impairment. Although a minor improvement in cognitive abilities has been noted, secondary dysfunction attributable to vascular-based cognitive decline remained. In terms of cognitive tolerance, derivatives of ketamine could potentially serve as an alternative to electroconvulsive therapy in cases of treatment-resistant depression, potentially improving short-term cognitive outcomes.

**Disclosure of Interest:**

None Declared

